# Seeking justice amidst chaos: methods to identify and document individuals implicated in crimes against the Rohingya in August 2017

**DOI:** 10.1186/s13031-022-00438-0

**Published:** 2022-03-02

**Authors:** Jennifer Leigh, Alexander Blum, Agnes Petty, Andrea Woods, Parveen Parmar, Chris Beyrer

**Affiliations:** 1 Physicians for Human Rights, New York, NY USA; 2 Johns Hopkins Bloomberg School of Public Health, Baltimore, MD USA; 3 Johns Hopkins University School of Medicine, Baltimore, MD USA; 4 Clinical Emergency Medicine, University of Southern California, Los Angeles, CA USA

**Keywords:** Human rights, Perpetrators, Accountability, Rohingya, Myanmar, Methodology

## Abstract

**Background:**

Documenting perpetrators of human rights violations enables effective prosecution and can help prevent future atrocities. Doing so calls for collecting reliable data using verifiable and transparent methodology. We present methods used to document crimes and identify alleged perpetrators implicated in the 2017 attacks against Rohingya civilians in Myanmar. The findings and lessons-learned have relevance to contemporary crises with widespread atrocities.

**Methods:**

A mixed-methods assessment conducted from May to July 2018 included: (1) cross-sectional quantitative surveys among leaders of affected hamlets in northern Rakhine State, (2) qualitative interviews to record hamlet-level accounts, and (3) clinical evaluations of survivors of violence. Survey respondents who reported violence and destruction in each hamlet were asked to identify perpetrators of those acts, including known role or affiliation. The reported names were reviewed for clarity and divergent spellings, repeated references were aggregated, and the names and roles were analyzed and classified by location and affiliation.

**Results:**

143 individuals were implicated in atrocities committed across three Northern Rakhine townships. Each was independently identified by at least three separate survey respondents as directly committing violence or destruction in their hamlet of origin, or as witnessed while fleeing to Bangladesh. Two-thirds (69%) of identified perpetrators were reported by four or more participants and 47% by five or more. Some form of additional identifying information, was provided for 85% of names. The most common affiliations were: Myanmar army (n = 40), Border Guard Police (n = 32), Village Tract Administrators (n = 17), and extremists (n = 25).

**Conclusions:**

The methodology presented here yielded a unique record of individuals purported to have directly committed acts of violence and destruction in Rakhine State in August 2017, forming the most extensive record of individuals implicated in ground-level perpetration of those crimes. This methodology can play a key role in accountability mechanisms for the Rohingya, and in other settings in which perpetrators are many and documentation of their crimes is difficult. The use of survey methods and standardized data collection amongst affected populations to comprehensively characterize crimes committed and to identify individuals implicated in those crimes can serve as a key tool in documentation and an important component of accountability.

## Introduction

Documented identification of individuals responsible for perpetrating human rights violations enables effective prosecution and can help prevent future atrocities [1]. Perpetrator identification is a crucial step in the justice and reconciliation process, and has been used successfully in Rwanda and Cambodia to alleviate trauma in the aftermath of genocidal violence [2, 3]. Individuals named through these processes may also provide important testimonies relevant to crimes being tried by international tribunals, which can strengthen efforts to hold governments and individuals accountable, create a shared understanding of the events that transpired and the crimes committed, and thereby facilitate restorative justice for the victims and their communities [4].

Documenting human rights violations to advocate for justice calls for reliable data and the use of verifiable and transparent methodology [5]. To this end, human rights research often emphasizes the use of highly standardized methods and, where possible, probability-based sampling, consistency across data collection methods and assessors, and intensive quality assurance and quality control measures. While some standardized tools exist, such as those produced by Human Rights Information and Documentation Systems and the Public International Law & Policy Group, there are few existing methodologies that can be readily implemented within the context of systematic quantitative surveys [6, 7]. The Office of the High Commissioner for Human Rights (OHCHR) provides guidance on human rights data collection and relevant indicators and the World Health Organization (WHO) offers ethical and safety recommendations for researching and documenting sexual violence in emergencies.

Effectively translating guidance and frameworks from OHCHR, WHO, and other organizations is rarely straightforward in conflict zones. Settings of acute crisis present unique barriers to implementing the standardized research methods that traditionally characterize surveys, interviews, and documentation of affected populations. The barriers to such methods in conflict-affected settings are simultaneously logistical, structural, environmental, cultural, and ethical. Research teams often face challenges in safely accessing displaced populations in conflict-affected areas and ensuring the safety of participants. These are exacerbated by environmental challenges, such as rainy seasons and hazardous conditions that hinder travel to remote settings where displaced populations often reside. Mandatory legal permits and restrictive access mediated by gatekeepers and authority figures add additional structural complexity, particularly if the host government has concerns about human rights research.

In addition to these standard challenges, faced across humanitarian research, ethical concerns add an additional layer of consideration and complexity. In the documentation of human rights violations, the need for careful documentation must be balanced with minimizing psychosocial harm, stigma, safety, and potential violence to participants in human rights assessments.

Like other settings where atrocities have been committed, these challenges arose in documentation and efforts to seek justice and accountability for the 2017 attacks against Rohingya civilians in northern Rakhine State. The Rohingya represent one of the largest stateless populations in the world, having experienced decades of discrimination, marginalization, and citizenship stripping in Myanmar [8]. This structural violence progressed to become physical attacks on the community in 2012 and 2016, which sent waves of Rohingya to seek refuge in neighboring Muslim-majority countries, namely Bangladesh, Malaysia, and Pakistan.

On August 25, 2017, the Arakan Rohingya Salvation Army (ARSA), an independent ethnic armed group, claimed responsibility for attacks on 30 police and military posts in northern Rakhine State that killed 12 Myanmar security personnel. Following the ARSA offensive, the Myanmar armed forces (collectively called the Tatmadaw) and Border Guard Police (BGP) initiated a widespread indiscriminate campaign of violence against Rohingya civilians across northern Rakhine State. The immediacy of the campaign and the coordination of the forces deployed undercut the Myanmar government’s characterization of the actions as a “response.” Independent investigations by human rights organizations suggest that the campaign was a premeditated clearance operation that utilized forces and equipment pre-emptively relocated to northern Rakhine state in anticipation of an opportunity to aggressively deploy [9–11]. The violence resulted in the death of approximately 7800 Rohingya people and mass displacement of survivors [12, 13]. Around 700,000 Rohingya refugees fled to Bangladesh, joining 300,000 refugees already living in previously established refugee camps at Kutupalong and Nayapara, just inside Bangladesh in Cox’s Bazar District. Despite the extensive documentation of the August 2017 violence, few individuals have been named as perpetrators. Those identified have largely been command-level authorities rather than ground-level individuals who directly perpetrated violence.

Drawing on data collected as part of a larger survey documenting the 2017 violence and resultant mortality, this study sought to identify individuals who allegedly carried out violence and were subsequently named by Rohingya community members. The analysis presented here provides a comprehensive methodology used to document crimes and identify alleged perpetrators. We hope that our findings can both contribute to efforts to secure justice for the Rohingya, as well as provide a methodology appropriate for other austere environments of crisis in which populations have suffered widespread atrocities.

## Methods

Data for this analysis are derived from a larger human rights assessment conducted by Physicians for Human Rights (PHR) to estimate the scale of violence against Rohingya civilians in northern Rakhine State in August 2017 and associated morbidity and mortality. Detailed methodology on all three study arms has been previously published in peer-reviewed literature [12, 14, 15] as well as in reports submitted to the United Nations Human Rights Council (UNHCR) [16]. This analysis draws from the quantitative arm of the study.

### Design

The assessment utilized a mixed-methods approach with a three arms design: (1) cross-sectional quantitative surveys among leaders of affected hamlets from all three townships of northern Rakhine State, (2) qualitative interviews documenting village-level narratives, and (3) clinical evaluations of survivors to medically corroborate their narratives. Leaders participating in the quantitative survey who reported 10 or more deaths in a single hamlet, the occurrence of mass rape, and/or the presence of a mass grave were invited to participate in subsequent in-depth interviews (arm 2) for detailed documentation of the crimes that occurred in their communities [15]. The forensic evaluation arm documented physical injuries sustained by select Rohingya survivors and corroborated findings with their accounts, including geographic location and type of violence [14]. Data reported here are drawn from the quantitative survey results [12], though are connected to data collected through the other methodologies.

### Survey sample

604 displaced Rohingya leaders from 590 hamlets^1^ and 12 urban wards in northern Rakhine State were surveyed from May to July 2018 to capture the scale and scope of the August 2017 violence, to characterize the nature of human rights violations, to document the perpetrators of those violations, and to quantify the mortality that stemmed from the attacks. To establish the study population, we used data from the Myanmar Information Management Unit, NGOs that had operated in northern Rakhine State, and community partners to generate a list of all hamlets in northern Rakhine State with Rohingya occupants. A leader from each of those hamlets was subsequently sought as a respondent from amongst the Rohingya present in Bangladesh. Our sample of 604 leaders thus included representatives from all or nearly all hamlets with Rohingya residents in northern Rakhine State (and constituted approximately two thirds of the 912 total villages reported in northern Rakhine State at that time). The hamlets were distributed roughly proportionately across the three townships of northern Rakhine State. The village leaders were asked to speak to the experience of the hamlet as a whole. The Myanmar government requires that Rohingya hamlet leaders regularly report population data for each hamlet, which positioned them well to share hamlet-level population estimates.

### Survey measures and implementation

The quantitative survey was electronically programmed for interviewer administered data collection and took 30 min to an hour to complete. Survey measures spanned the following domains: respondent and hamlet characteristics; reasons for flight from northern Rakhine State; meetings and arrests leading up to the events of August 2017; the types of violence experienced by members of the hamlet; the types of destruction that occurred in the hamlet, if any; violence which occurred during flight; and perpetrators.

The Rohingya language does not currently have a written form, and there is no standard method of transliteration into Burmese or English script; thus, the survey was developed in English and translated by a Rohingya survey team member and transliterated using English script. The survey team was then trained using the transliteration to administer the survey orally.

Respondents were given the option to refuse any survey question. Surveys were conducted in private to minimize risk of bias and security risk to the participant. Recall bias was minimized by a two-step process: on the day that participants consented to be interviewed, they were informed about the survey content and detail of questions and asked to think about and prepare to answer the questions. Data collectors returned one to two days later to conduct the survey, giving participants time to think about dates, types of violence, and individuals implicated in the attacks, rather than expecting participants to perform thorough complex cognitive recall processes to answer survey measures on short notice.

Following documentation of violence and destruction in each hamlet, respondents were asked about the perpetrators of those acts. Each respondent was asked to identify the affiliations of individuals that they witnessed perpetrating violence or destruction from a list of 31 categories (e.g. military, Border Guard Police, Village Tract Administrators), followed by an open-ended field for naming specific individuals. Respondents were asked to list as many names as possible and asked to identify their role or affiliation.

### Identification and classification of alleged perpetrators

The analytic team reviewed the qualitative text entries of names and roles of alleged perpetrators and created a comprehensive list of initial entries. Entries that did not refer to a specific individual, such as “BGP cadet” or “monks” were omitted from the list. Entries with both the same spelling and same affiliation were then aggregated. Names recorded using common diminutive forms, such as Mg for Maung and Ag for Aung were also aggregated where appropriate. The analytic team worked with native Burmese and Rohingya speakers to further aggregate entries with spellings that were similar, but slightly variant, due to variability in transliteration, such as duplicate letters or letter substitution (e.g., “Zay Ya” and “Zaya,” “So” and “Soe”).

The process of identifying perpetrators of atrocities is not without risk of misidentification. In this context, there is also risk of inadvertent conflation of different individuals with similar names, due to the traditional naming system of the Buddhist Bamar majority, which comprises a significant proportion of Tatmadaw personnel. The traditional Bamar naming system is astrology-based, and names are crafted from an established syllabary scheme determined by the individual’s day and time of birth [17]. As a result, there is often significant overlap in name permutations across the Bamar population. Bamar names also lack surnames, which further complicates the serial tracing and identification of individuals [17].

To mitigate risk of misidentification, we applied the following criteria to create a list for external validation and review: (1) If names were determined to be the same, but the associated identifying information (i.e. role) was inconsistent, then the instances were treated as different individuals; (2) The respondent’s hamlet of origin was also considered in the context of geographical proximity. If respondents reporting the same name were from hamlets or village tracts far from one another, the name was considered to refer to two separate individuals, unless the additional identifying information was very strongly consistent (such as both reporting the individual to be an officer in a specific army unit). This approach was informed by previously well-established norms of military units carrying out the 2017 Rakhine State clearance campaigns in a localized manner; and (3) to further minimize misidentifications or coincidental repeats, individuals were only included in the final list if they were uniquely named by at least three separate survey respondents.

The list was then reviewed by an internal team of native Rohingya speakers, experts in human rights research, and other experts with knowledge of the Myanmar military and BGP structure that were active during the 2017 clearance operations. This enabled the team to correctly associate individuals with specific regiments, and to correctly attribute acronyms and abbreviations that were associated with reported names. They also provided insight into the structure of Myanmar military and BGP units active in Rakhine State during the 2017 clearance operations.

This produced a final list of alleged perpetrators and their affiliations for external review that was stratified by township and major affiliation groups.

### External validation

A literature review of documents reporting on the 2017 violence was conducted to cross-reference other sources naming perpetrators and to further validate our findings. An initial group of documents were collected from human rights organizations and other longitudinally engaged organizations known to have reported on the violence against the Rohingya. These included Amnesty International, British Broadcasting Corporation (BBC), Fortify Rights, Human Rights Watch, the Independent International Fact-Finding Mission on Myanmar (IIMM), Médecins Sans Frontières (MSF), Physicians for Human Rights (PHR), Public Interest Law and Policy Group, and Reuters. Additional searches were conducted using PubMed, Lexis Nexis, and Google. Additional publications were identified through citation mining. In all, we reviewed 426 documents, eight of which identified individuals alleged to have perpetrated human rights violations (see Table 1). The literature review identified 42 named perpetrators, most of whom were higher-level personnel with authority inside the Myanmar army. Of these 42 perpetrators, six names overlapped with the list of individuals identified through the PHR assessment. In addition to the public documents, human rights partners were able to privately confirm overlap on eight additional names. Those names had been withheld from publication because they were not believed to be major perpetrators or did not meet those partner’s internal threshold for public identification.

**Table 1:** External sources identifying perpetrators in the August 2017 attacks on the Rohingya

Organization	Title	Publication date
Amnesty International	"We Will Destroy Everything” Military Responsibility for Crimes Against Humanity in Rakhine State, Myanmar	June 2018
Fortify Rights	"They Gave Them Long Swords" Preparations for Genocide and Crimes Against Humanity Against Rohingya Muslims in Rakhine State, Myanmar	July 2018
Fortify Rights & Human Rights Watch	"Joint Submission to CEDAW on Myanmar"	May 2018
Human Rights Watch	"Crimes against Humanity by Burmese Security Forces Against the Rohingya Muslim Population in Northern Rakhine State since August 25, 2017"	September 2017
Human Rights Watch	"Burma: Military Massacres Dozens in Rohingya Village"	October 2017
New York Times	“For Rohingya, Years of Torture at the Hands of a Neighbor”	August 2018
Reuters	"The shock troops who expelled the Rohingya from Myanmar"	June 2018
Reuters	"Myanmar Burning Series: Massacre in Myanmar"	February 2018

### Ethical considerations

The PHR Ethics Review Board provided ethical approval for this study. Because no formal Rohingya body exists that could serve as a review board, PHR held a community consultation with Rohingya leadership before administration of the qualitative and quantitative components of this work, to obtain their input, feedback, and approval. All study participants underwent a verbal informed consent process in the Rohingya language before participating in this study.

Due to the sensitive nature of the data collected, the study team undertook special efforts to prioritize the safety of survey participants. Specifically, identifying characteristics of the individuals who named the alleged perpetrators are not presented here, due to the real threat of reprisal from the Myanmar government or military following any repatriation efforts to their native homeland in northern Rakhine State. Data collectors did not record any identifying information on respondents beyond their hamlet of origin and leadership role.

## Results

Our analysis found 143 individuals implicated in atrocities committed across the three northern Rakhine townships. Each individual named was reported by at least three separate Rohingya respondents for directly committing violence or destroying property in the respondent’s hamlet of origin, or as witnessed by them during their flight to Bangladesh. Two-thirds (69%) of names were reported by four or more respondents, 47% by five or more, and 10% by more than ten respondents. Out of 604 total survey respondents, 324 reported information on specific individuals (54%). Affiliations, or some form of additional identifying information, was provided for 85% of names. The most common affiliations were the Myanmar Army, the BGP, Village Tract Administrators (VTA), and Rakhine extremists (see Table 2).

**Table 2:** Affiliations and locations of perpetrators alleged to have perpetrated crimes in the 2017 attacks on the Rohingya of northern Rakhine State

	Army*	BGP	VTA	Extremists	Other/unknown	Total^+^
Buthidaung	32	16	6	8	20	81
Maungdaw	16	13	6	12	12	59
Rathedaung	3	3	5	5	0	14
Total	40	32	17	25	32	143

*11 Individuals affiliated with the army operated in both Buthidaung and Maungdaw and are therefore included in the number for both Townships, but are not double counted in the totals

^+^3 of the VTAs were also named as extremists, and thus the total of the five categories is less and has been reduced accordingly

The affiliations reported for the 143 individuals named were consistent with the perpetrator groups identified through the close-ended question on the survey identifying major perpetrator classifications. As with individual affiliations, the army and BGP were the groups most frequently reported, followed by Rakhine extremists and local government officials, including VTAs. The proportion of respondents implicating groups in violence in the hamlets was similar to the proportion implicating them in violence during flight, except for Rakhine extremists and Rakhine State militia, both of which were more likely to have been reported for violence during flight (Table 3).

**Table 3:** Percent of quantitative survey respondents reporting each group’s participation in violence and destruction in northern Rakhine State

	In the hamlet (%)	While fleeing to Bangladesh (%)
Border Guard Police	90.4	88.7
Military/Tatmadaw	87.2	88.7
33rd Light Infantry Division	27.8	26.3
Regiment 552	25.6	27.4
Regiment 564	16.6	13.4
Regiment 551	15.3	10.2
Rakhine extremists	66.7	77.5
969 members^+^	13.9	13.1
Local government officials*	56.5	56.3
Rakhine state militias	27.1	39.4
Sakma extremists	14.5	31.3
Hindu extremists	12.4	10.2

*Including “civil government,” “Village Tract Administrators,” and “District and Township Administrators”

^+^969 is a Buddhist monk-led nationalistic movement advocating intolerance toward Muslims

**Fig. 1:**
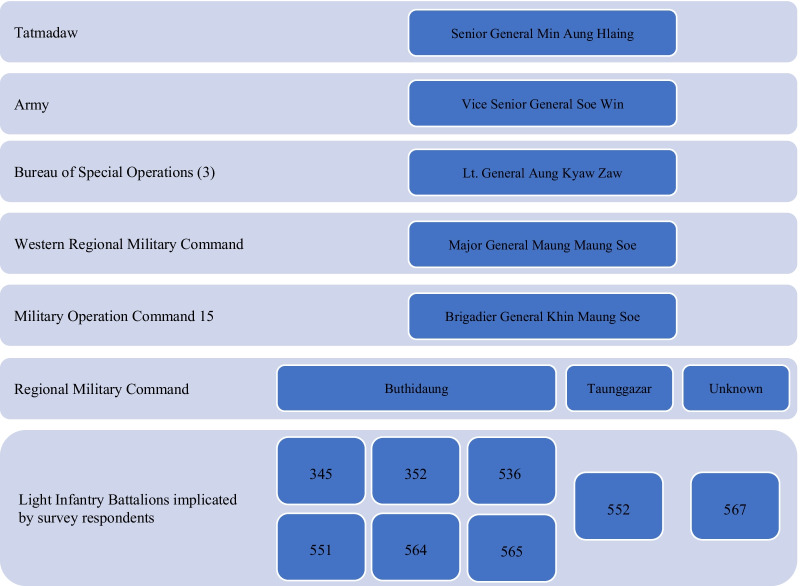
Command structure of Tatmadaw units named by respondents as involved in the August 2017 violence. *Sources*: “Command Structure of the Myanmar Army’s Operation in Rakhine,” 2017; The Structure and Operations of the Myanmar Army in Rakhine State, 2018; “We Will Destroy Everything”, 2018; Independent International Fact-Finding Mission on Myanmar, 2018; “They Gave Them Long Swords,” 2018

The Myanmar army was the most frequently identified affiliation of implicated individuals. Forty individuals named as perpetrators were identified as members of the Myanmar army. The army is the largest branch of Myanmar’s armed forces. Within the army, operations in northern Rakhine State are overseen by the Western Regional Military Command (WRMC) (Fig. 1). The individuals named include personnel from eight Light Infantry Battalions (LIBs): 345, 352, 536, 537, 551, 552, 564, and 565. Of those affiliated with LIBs, Commanding Officers were named for five of the eight battalions, as were a number of other officers and their ranks. All eight LIBs fall under Military Operation Command (MOC) 15, which is part of the WRMC. One officer from Light Infantry Division (LID) 33 was also implicated. For seven individuals, their unit affiliation was unknown or unclear.

In addition to the army units implicated, survey respondents also identified four individuals as members of local militias, primarily comprised of Buddhists from the majority Rakhine (also known as Arakanese) ethnic group.

Thirty-two individuals identified as perpetrators were described as members of the BGP from nine different sectors. Of those, 20 were affiliated with a specific sector, and for 12, their sector affiliation was unknown or unclear. Several of the reported names were associated with two separate sector numbers. This derived from the change in the designated numbers for these sectors. From 1992, the area was administered by the Border Administration Force, known as the Na Sa Ka based on its Burmese acronym. The sectors were renumbered in 2013 when Na Sa Ka was repealed and the BGP took over command. Many people still refer to areas by their former sector numbers, resulting in potential confusion. With input from Rohingya colleagues, the former Na Sa Ka numbers were aligned with the current BGP numbers, so that if a respondent was reported to be associated with two different sectors, it was readily checked whether the two numbers in fact referred to the same area. For example, the area around Taung Bazar was Na Sa Ka Sector 9, but for BGP the same area is Sector 10, subsector 20.

Ward administrators and VTAs are local-level officials who serve as the primary intermediaries between civilians and the government of Myanmar. Seventeen individuals identified as perpetrators were named as VTAs in village tracts across all three townships. Most were serving as VTAs in August 2017, but several were identified as former VTAs, suggesting that they had ended their terms prior to August 2017.

Twenty-five individuals named as perpetrators were identified by respondents to be “Rakhine extremists,” a term that refers to individuals widely known to be anti-Muslim or belonging to anti-Muslim organizations. Five were specifically listed as being 969 members, referring to a Buddhist monk-led nationalistic movement, also known as Ma Ba Tha, advocating intolerance toward Muslims and boycott of Muslim-owned businesses [18]. Two of these individuals were named as current VTAs and one as a previous VTA, indicating that identified extremists were members of local government. Six of the individuals were identified specifically as Hindu extremists, alluding to Hindu residents of northern Rakhine State who are also anti-Muslim or anti-Rohingya.

In addition to the 115 names associated with the four categories above, another 28 names were implicated as perpetrators with other, unclear, or no affiliations given. These individuals were from Buthidaung [16] and Maungdaw [12]. Only six included any further additional identifying information, such as “chairman,” “cadet,” or “Sa Ya Hpa” (Central Intelligence Department).

## Discussion

The names collected via this methodology comprise a unique record of individuals purported to have directly participated in committing acts of physical, psychological, and sexual violence and destruction of property in northern Rakhine State in August 2017. Each recorded name was submitted by individuals who directly witnessed or had firsthand knowledge of the named perpetrator’s crime(s). While other sources have provided extensive documentation of a limited number of individuals, primarily involved in higher-level planning and coordination of the attacks, our methodology focused more specifically on identifying frontline perpetrators. Our data forms the most extensive record of individuals implicated in ground-level perpetration of crimes in the 2017 northern Rakhine State clearance campaigns.

To maximize the utility of this information to ongoing international accountability efforts, we maintained open channels of communication with PHR throughout the analysis process to establish shared goals and expectations. Coordination and integration were guiding principles as we sought to consolidate our findings into existing narratives and prosecutorial frameworks. Being mindful of and patient with organizational constraints and legal boundaries is crucial to maintaining collaborative partnerships and to maximizing shared impact.

To that end, PHR has subsequently shared the list of 143 alleged perpetrators and their affiliations with international accountability mechanisms, for use in their investigations and case building. Accountability mechanisms such as the UN’s Independent Investigative Mechanism for Myanmar, the International Court of Justice, and the International Criminal Court are well suited to triangulate sensitive, unpublished data collected by multiple human rights investigations to further validate and identify individuals implicated in crimes under investigation.

While we do not expect the International Criminal Court (ICC) to bring charges against each of the individuals named through this process, these data are nonetheless a valuable contribution towards accountability of those in leadership positions. These types of cases—including one filed by The Gambia against Myanmar [19]—are often pursued in the ICC or the International Court of Justice. More broadly, systematically documenting perpetrators can provide insight into the scale of atrocities committed, while also supporting cases against higher-level authorities [20]. In the absence of international intervention in settings of humanitarian crisis, prompt identification of alleged perpetrators and documentation of their crimes is crucial to understanding the events that transpired, and to supporting some semblance of justice for the victimized population. Above all, pursuing this work aligns well with universal humanitarian ideals of truth-telling, healing, and accountability that are valuable in any setting.

In a country such as Myanmar, where national armed forces regularly commit widespread human rights violations against civilians, methodical documentation of perpetrators also provides local and international observers with the ability to project the severity of violence based on security personnel deployed to a conflict zone [21–23]. The nationwide crackdown on peaceful protests following the February 2021 coup d’etat in Myanmar is one such example. The arrival of the LID, given their record of involvement in military clearance operations in Rakhine and Shan States, warned protesters and human rights watchdogs alike of imminent escalations in violence [24, 25]. Indeed, examination of subsequent media footage implicated several LIDs—including the 33rd—in indiscriminate and excessive use of force and lethal battlefield weaponry against unarmed protesters [26]. Notably, Min Aung Hlaing was the head of the command structure for both the 2017 Rohingya violence and the 2021 coup.

The names of the alleged perpetrators are not included here because—apart from six names also reported by external sources—they have not been confirmed by other means of verification. Although all the individuals included in our final list were independently cited by at least three separate respondents, the names were gathered by a single means of data collection. None of the alleged perpetrators were subsequently contacted or spoken to. Therefore, while these names present a very good starting point for further investigation, inclusion in the list alone is insufficient to unambiguously confirm any individual’s role as a perpetrator. Furthermore, there exists a possibility of reprisal against those named, whether by Rohingya remaining in northern Rakhine State or by others, and thus reporting unconfirmed names here poses a risk that is unacceptable in the case of individuals who may have been mistakenly identified.

We do not believe that these limitations compromise the legitimacy or authenticity of our list. Rather, this contextual acknowledgement underscores the nature of this list as an organized and collated report of individuals named by the Rohingya and not the product of any subsequent investigation or inquiry. Further validation of these data by other means is welcomed.

The potential limitations of the data described above are balanced by several strengths that are derived from the methodological rigor of the assessment, as well as the depth and breadth of the information provided by participants. First, the frequency with which certain individuals were independently named as alleged perpetrators by three or more survey respondents and their associated affiliations are consistent with extensively documented narrative accounts of the events of August 2017 [10, 27, 28]. Our data adds an additional level of insight into the mechanics of collaboration among the military, BGP, VTAs, and civilian extremists to carry out clearance operations against the Rohingya. Almost two-thirds (65%) of individuals identified through our analysis were affiliated with the military, BGP, or VTA positions. Thus, these individuals have explicit and official ties to—and were acting under the authority of—the government of Myanmar.

This analysis was further strengthened through a comprehensive review of existing literature that identified individuals and military units active in specific locales. Based on associations and military unit affiliations linked to each perpetrator from the original data collected in interviews, we were able to more meaningfully integrate the names generated by our analysis into pre-existing frameworks already being used to pursue accountability in court. We believe that doing so maximizes the utility of ground-level perpetrator data and provides valuable insight into the mechanics of how atrocities committed against the Rohingya were carried out in a systematic manner within linear chains of military command and local governance frameworks. This level of detail fills in gaps that could otherwise be exploited in court when trying to prove culpability of military commanders and other leadership figures. Though this list of names undoubtedly does not include every ground-level perpetrator, the fractional representation of these different groups among the 143 total names reinforces the well-established understanding that the government of Myanmar leveraged its military, police, and local governance assets to carry out widespread systematic attacks against the Rohingya in northern Rakhine State. The quantitative findings underline this, with the majority of respondents reporting the participation of government forces, including the BGP (90%), the Tatmadaw (87%), and VTAs (57%).

The methodology presented here can serve as a model for identification and documentation of human rights violations and those alleged to have perpetrated such crimes in other settings. Our findings suggest there is value added by including standardized open-ended questions to identify individual perpetrators when conducting surveys documenting human rights abuses. Doing so through private interviewer-administered surveys can elicit detailed information and minimize groupthink mentality that may otherwise unduly bias responses. Collecting this information as part of broader surveys, rather than solely through traditional methods of in-depth interviews among purposively sampled individuals, maximizes internal and external validity of data.

One challenge that we encountered—and anticipate would be common to other settings of international humanitarian conflict—was reconciling variabilities in spelling of alleged perpetrator names. Minor differences are often attributable to slight variations in respondent pronunciation and interviewer transcription into written text using the English alphabet. In our experience, any discrepancies in the raw data must be addressed on a case-by-case basis prior to final analysis to avoid inaccurate assumptions.

In our case, this was particularly challenging because the Rohingya language is an oral tradition with no formal written script; local dialects often include components of Burmese as well. These linguistic nuances underlined the importance of including native speakers and those with expertise in local dialects in the analytic process, so we consulted Burmese and Rohingya speakers accordingly. Doing so helped reconcile discrepancies, eliminate redundancies, and clarify uncertainties. More broadly, cross referencing findings with those of other human rights organizations provides another level of corroboration, while also contributing to a shared spirit of collaboration and cooperation necessary to build the strongest case possible for accountability of perpetrators. Nonetheless, when in doubt, we erred conservatively in not combining similar yet non-identical names, optimizing our confidence in the final list of alleged perpetrators.

Reflecting on the course of our research, we have identified several recommendations oriented towards ensuring that human rights data is collected and analyzed as rigorously as possible for accountability mechanisms and as evidence for prosecution. The primary objectives of our assessment tool were to capture the scale of violence and destruction perpetrated against Rohingya communities in northern Rakhine State in 2017. Our mixed methods research design was structured to align with those objectives and secondarily collected names of individuals alleged to have perpetrated crimes that occurred during these attacks. The assessment tool was designed to assess scale and mortality and balanced depth and breadth of data against time burden, participant fatigue, and other logistical challenges. We provide the following recommendations for investigations that primarily aim to identify individual crimes and those alleged to have committed them.

First, we suggest further standardizing the collection of perpetrator information. In our study, respondents were asked to describe (1) types of violence; (2) whether they witnessed the violence, heard about it, or both; and (3) the category/association of perpetrators who committed the described acts. Afterwards, there was an open-ended opportunity to share the names of any individuals who they believed were involved in perpetrating violence. The tool was intentionally designed this way, as its primary purpose was to estimate the prevalence of violence, destruction, and mortality. Identifying perpetrators from the information collected was secondary. However, the survey’s design meant that any names shared by respondents were not explicitly linked to any of the prior question responses, which often consisted of multi-item lists shared by respondents. It therefore became difficult to discern which—if any—of the names provided were associated with specific crimes or categories/associations from the respondent’s prior responses.

For surveys designed with the primary goal of collecting perpetrator information, we recommend providing explicit prompts to gather more information about each individually named perpetrator. This could include roles and affiliations, location of the crime, and whether the alleged perpetrator directly implemented violence, ordered it, or observed it. Lists can be iteratively generated and refined through regular reviews of collected data and debriefing with surveyors at short intervals (i.e. weekly). Check-ins also aid in clarification of responses, elaboration of unfamiliar acronyms and abbreviations, and standardization of spelling. This is often logistically challenging, particularly in emergency settings, but doing so minimizes challenges with variability in data entry and thus mitigates uncertainty in subsequent analysis. Maintaining open channels of secure communication through messaging services such as WhatsApp or Signal is similarly important for resolving any issues that arise in the field.

In our experience interfacing with lawyers and prosecutors, we learned that initial accounts and allegations—such as those captured by broad level survey questions—are often strengthened by follow-up and further conversation when being considered for presentation as formal evidence in court. We therefore recommend asking respondents at the time of interview if they would be willing to be contacted by prosecutorial investigation teams in the future. Mindful of the need for confidentiality, names and contact information should follow standard data security practices and should be retained apart from survey data, which are identified only by unique codes [29]. Of equal importance is an honest recognition that accurate, thorough follow-up with respondents in conflict settings and humanitarian crises is an unusually challenging endeavor. These populations often lack reliable access to telephones, internet, stable housing, identification cards, and many of the other basic resources that are crucial to successful re-contact months or years after an initial interview. Witnesses’ living circumstances and ability to be located and contacted are highly variable, which diminishes the likelihood of successfully re-establishing contact for further investigation.

Ultimately, embracing an inter-disciplinary approach by embedding legal or prosecutorial experts into survey teams during the original interviews could be a more feasible alternative to recontacting witnesses. This would allow for collection of evidence that could be used in court without necessitating re-establishment of contact.

Finally, recording additional details regarding the acts committed by the individuals named would be useful, but a balance must be discerned between standardized questionnaires that facilitate quantitative analysis and allowing for flexibility in individual narratives. Additional details are valuable for understanding the distribution of crimes carried out by individuals affiliated with different entities, such as the military, police, or local governance. Elaboration also provides some characterization of the wrongdoing committed. Without this information, it becomes impossible to know if a named perpetrator burned a family’s home or took a human life, which are objectively different crimes. At the same time, foregoing any form of standardization in collecting broad-scale perpetrator information introduces logistical challenges in meaningfully capturing and analyzing narrative-style accounts. Free form interviews are also likely to introduce additional challenges around language and translation.

**Fig. 2:**
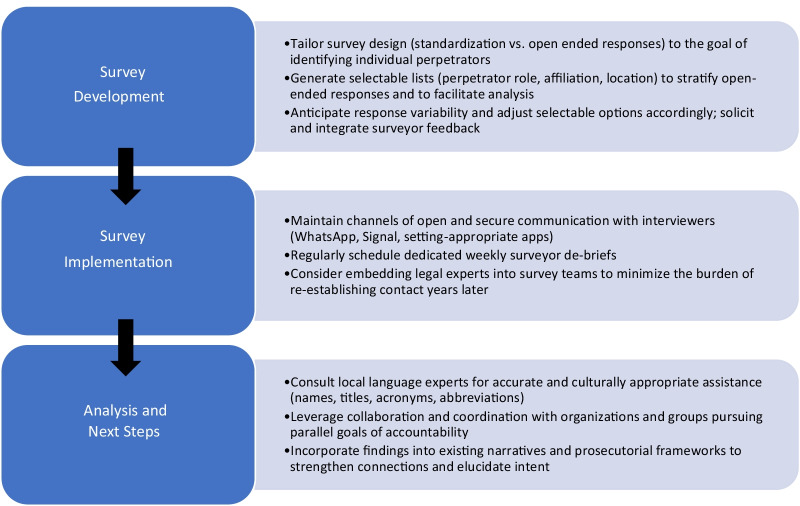
Summary of recommendations for developing and implementing surveys intended to capture ground-level perpetrator information in settings of humanitarian conflict

One way to reconcile these competing dynamics is to generate selectable lists that explicitly link the most common affiliations (i.e., police, military, extremists) and crimes (i.e., arson, murder, sexual assault, looting, etc.) to individually named perpetrators according to a given conflict’s circumstances and context. Including additional space for further commentary or details can help record information shared by respondents that cannot be adequately captured by the selectable lists of descriptors. Ultimately, it is a matter of the primary objectives of the investigation and balanced against time and cognitive burden of the participant in quantitative and qualitative interviews (Fig. 2).

## Conclusion

Recording the names of alleged ground-level perpetrators is an important component of accountability for the August 2017 crimes against the Rohingya. The Rohingya refugees whose experiences were documented through this survey and who participated in collecting this data were adamant that the international community be made aware of the events of August 2017 and that the individuals responsible be held accountable for the suffering and persecution that the Rohingya experienced.

We identified key architects of the August 2017 clearance operations, yielding findings consistent with several independent investigations led by other international human rights and health organizations. More significantly, our methodology also collected data on ground-level perpetrators who committed acts of physical, psychological, and sexual violence against the Rohingya. This ground-level information is critical in contexts such as Myanmar, where the country’s military forces are regularly deployed against civilians in a long-standing coordinated effort to establish ethnic hegemony. Careful monitoring of individual perpetrators by international observers and watchdog groups will be crucial to prevent further human rights violations against not only the Rohingya, but other ethnic minorities and vulnerable populations across Myanmar.

The names and affiliations documented make it clear that direct orders of retaliatory clearance operations were passed down from army and BGP leadership to multiple units, which carried them out by means of an extensive network of actors, including civilians and civil servants. This documentation of the ground-level individuals who have been implicated in acts of violence solidifies the direct link between command authority and frontline perpetrators. It also strengthens our understanding of the diverse range of personnel and groups aligned with the government of Myanmar that were brought together in a very intentional manner to carry out clearance operations against the Rohingya. Documented participation of individuals at the ground level is an important rung on the ladder of accountability.

The methodology presented here can play a key role in accountability mechanisms not only for the Rohingya, but in other settings where widespread atrocities have occurred and in which perpetrators are many and documentation of their involvement in crimes is difficult. Most international organizations, journalists, and foreign governments focus primarily on identifying the architects and leaders of human rights violations. These efforts are important and efficacious but can be strengthened by capturing information about ground-level perpetrators who are empowering those leaders to carry out population-level violence. Focusing solely on leadership figures does a disservice to all those who suffered at the hands of individual perpetrators who had at least some degree of agency in choosing to participate in wrongdoing. The use of survey methods and standardized data collection amongst affected populations to better characterize crimes committed and to identify individuals who are implicated in those crimes can serve as a key tool in documentation and an important component of accountability.

Recent events in South Sudan exemplify the value of having well-documented perpetrator-level information available for use in court. After prolonged delay by the country’s government, approval of a hybrid court intended to try individuals who committed the most serious crimes was granted in January 2021—more than seven years after the conflict first broke out in December 2013 [30]. Though it will be crucial for South Sudan to ensure the court’s legitimacy and transparency, it is also important to consider the quality of evidence that will be brought forth before its jurors. In these and other such circumstances, it becomes clear that gathering perpetrator-level data using the most robust methodologies that can reasonably be applied in settings of crisis plays a central in healing individuals and countries alike. Being especially thorough in methodology becomes particularly relevant when information is being scrutinized years or decades later, as is often the case with humanitarian crises. Pursuing accountability of human rights violators on the international stage through data collection and analysis is a challenging but worthwhile endeavor. We draw hope from Dr. Martin Luther King Jr’s. reminder that “the arc of the moral universe is long, but it bends towards justice.”

## Data Availability

The data that support the findings of this study are not publicly available due to concerns regarding the identification of respondents and the potential misidentification of alleged perpetrators. However, with appropriate redactions, the data are available from the corresponding author on reasonable request. Due to concerns about identification of respondents, hamlet level data will not be shared publicly. No identifying information on respondents was collected beyond their hamlet of origin and leadership role, however even that information may narrow the pool of potential respondents sufficiently to constitute a risk of identification, and consequently, the real threat of reprisal from the Myanmar government or military following any repatriation efforts to their native homeland in northern Rakhine State. Therefore, the research team has decided not to release any data at hamlet level which includes the hamlet name. Data may be shared at village tract level if hamlet names and respondent roles are redacted. Additionally, the names of the alleged perpetrators are not publicly available because they have not been confirmed by other means of verification. Although all the individuals included in our final list were independently cited by at least three separate respondents, the names were gathered by a single means of data collection. None of the alleged perpetrators were subsequently contacted or spoken to. Therefore, while these names present a very good starting point for further investigation, inclusion in the list alone is insufficient to unambiguously confirm any individual’s role as a perpetrator. Furthermore, there exists a possibility of reprisal against those named, whether by Rohingya remaining in northern Rakhine State or by others, and thus reporting unconfirmed names here poses a risk that is unacceptable in the case of individuals who may have been mistakenly identified. These names may be shared with accountability mechanisms, on a case by case basis.

## Abbreviations

ARSA: Arakan Rohingya Salvation Army
BBC: British Broadcasting Corporation
BGP: Border Guard Police
ICC: International Criminal Court
IIMM: Independent Investigative Mechanism for Myanmar
LIB: Light Infantry Battalion
LID: Light Infantry Division
MOC: Military Operation Command
MSF: Médecins Sans Frontières
NGO: Non-governmental Organization
OHCHR: Office of the High Commissioner for Human Rights
PHR: Physicians for Human Rights
UNHCR: United Nations Human Rights Council
VTA: Village Tract Administrator
WHO: World Health Organization
WRMC: Western Regional Military Command

## Footnotes

1 In Rakhine State, the term hamlet is equivalent to village and multiple hamlets comprise a larger administrative unit called a village tract. Multiple village tracts make up a township, of which there are three in the northern Rakhine State: Buthidaung, Maungdaw, and Rathedaung. The vast majority of Rohingya in Myanmar live in these three townships.
